# Molecular study on infection rates of *Anaplasma ovis* and *Anaplasma marginale* in sheep and cattle in West-Azerbaijan province, Iran

**Published:** 2016-06-15

**Authors:** Vahid Noaman, Davood Bastani

**Affiliations:** 1 *Veterinary Research Department, Isfahan Agriculture and Natural Resources Research and Education Center, AREEO , Isfahan, Iran; *; 2 *Graduate Student, Faculty of Veterinary Medicine, Islamic Azad University, Boroujerd Branch, Boroujerd, Iran.*

**Keywords:** *Anaplasma marginale*, *Anaplasma ovis*, Cattle, Sheep, West Azerbaijan

## Abstract

This study was carried out to determine the presence and frequency of *Anaplasma ovis and Anaplasma marginale *in sheep and dairy cattle in West-Azerbaijan province, Iran. A total number of 200 blood samples were randomly collected via the jugular vein from apparently healthy cattle (100) and sheep (100). The extracted DNA from blood cells was screened using genus-specific (*Anaplasma *spp*.*) nested-polymerase chain reaction (PCR) based on 16S rRNA gene primer sets. Species-specific PCR was set up using major surface protein 4 (MSP4) gene primer set. None of cattle blood samples were positive for *Anaplasma* spp. by the first nested PCR. Five samples among the 100 sheep blood samples were both positive in the first nested PCR and* A. ovis* -specific PCR, based on MSP4 gene. In total, 5.00% of animals were *A. ovis *positive. This study identified a low prevalence of *A. ovis* in the blood of apparently healthy sheep in West Azerbaijan province.

## Introduction

Anaplasmosis is an arthropod-borne disease of cattle and other ruminants which is caused by different geneses of *Anaplasma* species (Rickettsiales: *Anaplasmataceae*).^1^ Five Anaplasma genuses including *A. marginale*, *A. centrale*, *A. phagocytophilium*, *A. bovis* and *A. ovis* are usually identified in Iranian cattle and sheep using molecular methods (through blood sampling).^2^^-^^7^ *Anaplasma marginale* and *A. ovis* are erythrocytic parasites with similarity in morphology, biology and transmission by ixodid ticks and flies, however, they bear different capacity to infect their hosts. *A. marginale* is the main causative agent of bovine anaplasmosis and results in high economical loses in dairy cattle industry. In contrast,* A. ovis* does not cause severe disease, however, under some circumstances such as stress or other predisposing factors can induce anaplasmosis disease.^8^^-^^11^

Diagnosis of both *A. marginale* and *A. ovis* is performed routinely by their hosts and morphological identification based on location of inclusion bodies marginally within the erythrocytes.^12^ Giemsa-stained blood smears can be indeed used as a suitable method to detect *Anaplasma* agents in the animals clinically suspected to acute anaplasmosis, however, it is not applicable for the determination of pre-symptomatic and carrier animals.^13^

Serological tests like competitive enzyme-linked immunosorbent assay (cELISA) based on the major surface protein 5 (MSP5) and immunofluorescent antibody (IFA) test do not discriminate between different *Anaplasma* species because of antigenic similarity.^5^

Polymerase chain reaction (PCR) assay based on the 16S rRNA gene is invaluable technique for the detection of pathogenic bacteria that are difficult to isolate and grow in the laboratory. Amplification of 16S rRNA gene is commonly used for detection of *Anaplasma/Ehrlichia* genera, however, cannot differentiate *A. marginale, A. centrale and A. ovis* because of sequence similarity. For the first time, Noaman and Shayan designed PCR-restriction fragment length polymorphism (RFLP) test for detection and differentiation between *A. marginale*, *A. centrale* and *A. ovis* based on 16S rRNA in hosts.^3^ They reported that analysis of the 16S rRNA gene not only is useful for assisting to define genera but also for defining species.^5^ Nevertheless, the identification of the *Anaplasma* species by PCR-RFLP is a slow, time-consuming procedure and cumbersome for use in mass-screenings.^5^

 Six *A. marginale* major surface proteins (MSPs) have been identified and characterized. Three of them, MSP1a, MSP4 and MSP5, were encoded by single genes. MSP1a and MSP4 were used to characterize the genetic diversity of *Anaplasma *spp. The results confirmed that MSP1a is not a good marker for the characterization of geographic isolates of *A. marginale*, while use of MSP4 provides useful phylogeographic information.^14^

*Anaplasma marginale* and *A. ovis* have been investigated using molecular techniques in some parts of Iran.^2^^,^^15^^-^^18^ Although *Anaplasma* infection previously has been documented in Turkey and Iraq where are bordered by West-Azerbaijan province of Iran,^19^^,^^20^ there is very little information about the *Anaplasma* species in animals of West-Azerbaijan province.

Therefore, the purpose of this study was to assess knowledge about the presence and the prevalence of *A. marginale* and *A. ovis* in dairy cattle and sheep from West-Azerbaijan province.

## Materials and Methods

**Fig. 1:**
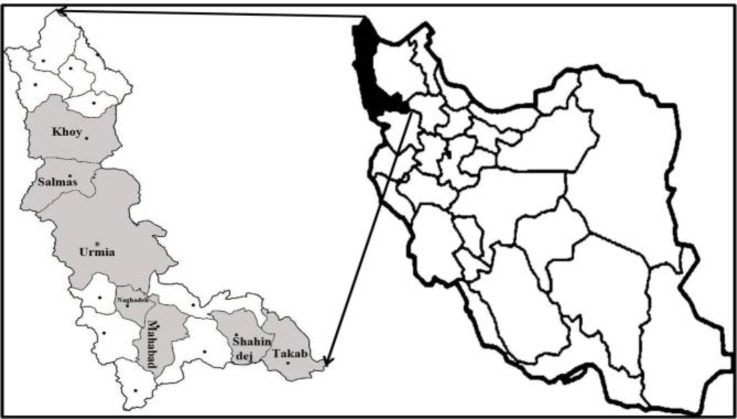
Geographic localization of the study area (West-Azerbaijan province. Iran

**Collection of blood samples.** The study was carried out on 20 cattle farms and 20 sheep flocks in seven regions of West-Azerbaijan province (latitude 35 ˚ 58′ to 39˚ 47′ N, longitude 44˚ 14′ to 47˚ 19′ E),^21^ Iran, during summer 2013 (Fig. 1). A total number of 200 blood samples were collected via the jugular vein from apparently healthy cattle (100) and sheep (100), randomly. In each farm or flock, samples were collected from five cows or sheep. The age of animals ranged from one to nine years. Blood samples were taken in tubes containing the anticoagulant ethylene diamine tetra-acetic acid (EDTA), (Ava Co., Tehran, Iran). The blood samples were stored at − 20 ˚C until DNA extraction.

**DNA extraction. **DNA of blood samples was extracted using a DNA extraction kit (Investigating group molecular biological system transfer, Tehran, Iran) according to the manufacturer's instructions.

**Nested-PCR. **The first PCR was performed using the universal primers fD1(5’-AGAGTTTGATCCTGGCTCAG-3’) and Rp2 (5’-ACGGCTACCTTGTTACGACTT-3’),^22^ in 50 μL total volume including 1X PCR buffer, 2.5 U Taq polymerase (SinaClon, Tehran, Iran), 2 μL of each primer (fD1/Rp2, 20 μM; SinaClon), 200 μM of each dATP, dTTP, dCTP and dGTP (SinaClon), 1.5 mM MgCl_2_ and 100-500 ng extracted DNA in automated thermocycler (Model T100; Bio-Rad, Hercules, USA) with the following program: 5 min incubation at 95 ˚C to denature double strand DNA, 40 cycles of 45 sec at 94 ˚C (denaturing step), 45 sec at 55 ˚C (annealing step) and 1.5 min, at 72 ˚C (extension step). Finally, PCR was completed with the additional extension step for 10 min. The PCR products were analyzed on 1% agarose gel (SinaClon) in 0.5X Tris-borate-EDTA (TBE) buffer and visualized using ethidium bromide (SinaClon) and UV-illuminator (TechnoGen, Tehran, Iran). For the nested PCR technique, primers EHR16SD (5’GGTACCTACAGAAGAAG TCC-3’) and EHR16SR (5’TAGCACTCATCGTT TACAGC-3’) were used to control the specificity of the PCR products for the 16S rRNA gene of *Anaplasma *spp.^23^ From the first PCR, 0.5 μL of product in a final volume of 25 μL was used in nested PCR with the following program: 5 min incubation at 95 ˚C to denature double strand DNA, 40 cycles of 45 sec at 94 ˚C (denaturing step), 45 sec at 60 ˚C (annealing step) and 1 min, at 72 ˚C (extension step). Finally, PCR stage was completed with the additional extension step for 10 min. The PCR products were analyzed on 1% agarose gel (SinaClon) in 0.5X Tris-borate-EDTA (TBE) buffer and visualized using ethidium bromide (SinaClon) and UV transilluminator (TechnoGen).

## Results

**Fig. 2:**
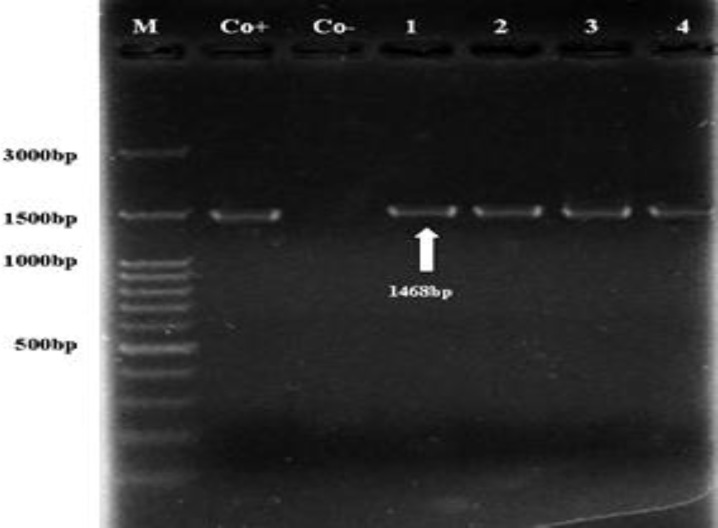
Primary amplification products based on 16S rRNA gene. The expected size (1468 bp) is indicated (lanes 1 to 4). Co+: Positive control. Co-: Negative control. M: Marker

The PCR analysis of the isolated DNA from 200 blood samples using primers fD1/ Rp2 revealed an expected PCR product with approximately 1468bp nucleotides in length from the 16S rRNA gene (Fig. 2). 

**Fig. 3.**
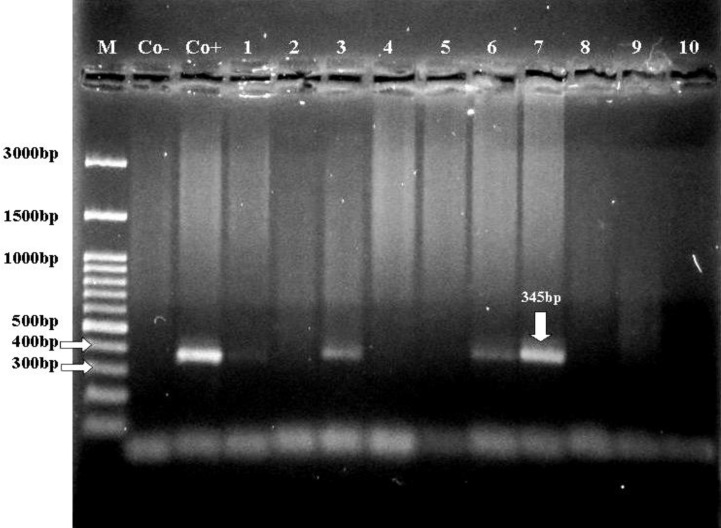
Nested amplification products based on 16S rRNA gene. The expected size (345 bp) is noted (lanes 1 to 7). Co+: Positive control. Co-: Negative control. M: Marker

To confirm the specificity of the first PCR products, the PCR products were amplified with the primers EHR16SD/EHR16SR, which were located between the fD1/Rp2 primers. Seven PCR products were amplified with the above mentioned primers (EHR16SD/EHR16SR), which denoted the first PCR product belonged to the 16S rRNA gene of* Anaplasma *spp. The amplified nested PCR product had an expected PCR product with 345 bp (Fig. 3). 

**Fig. 4:**
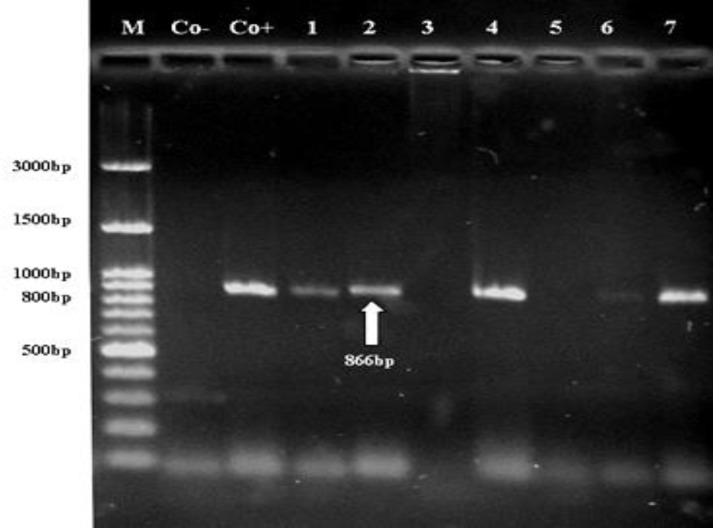
DNA amplification products based on MSP4 gene of *A. ovis*. The expected size (866 bp) is indicated (lanes 1, 2, 4, 6, and 7). Co+: Positive control. Co-: Negative control. M: Marker

The nested PCR were positively associated with only sheep samples. None of the bovine samples was ampliﬁed by the 16S rRNA PCR and nested PCR. Seven samples that were positive using nested PCR technique were ampliﬁed by the MSP45/MSP43 primers based on MSP4 gene. ^24^ Five samples were positive for *A. ovis* by RCR based on MSP4 and had an expected PCR product with 866 bp (Fig. 4). Complementary tests should be carried out to detect two unknown sheep samples.

## Discussion

The most common used method for diagnosis of *Anaplasma* infected cattle is the microscopic examination of Giemsa stained blood smears. But due to the low amount of parasitemia in carrier cattle and the difficulty to differentiate between *Anaplasma* organism and other structures like Heinz bodies, Howell-Jolly bodies or staining artifacts, which are often seen in Giemsa stained blood smears, this method is not recommended for the characterization of persistently infected cattle.^5^

Due to the very low amount of the *Anaplasma *infected erythrocytes in carrier animals and to enhance the sensitivity of *Anaplasma* PCR nested PCR from the16S rRNA gene of* Anaplasma *was performed.

For differentiation of *A. ovis *from *A. marginale*, the extracted DNA from positive nested PCR samples was amplified using MSP4 primers specific. ^24^

In the present study, none of the cattle samples was positive for *A. marginale* based on amplification of the MSP4 gene. In central part of Iran *A. marginale* 16S rRNA was detected in 38.60% of cattle without any clinical signs by PCR-RFLP.^2^ The lack of detection of *A. marginale* in the cattle samples can be explained mainly by the low samples in this study. However, more samples may be needed to define the prevalence of infection in the cattle of West-Azerbaijan province.

In the present study, *A. ovis* MSP4 amplicons were detected in 5.00% of sheep by PCR. Our previous findings detected *A. ovis* DNA in 33.33% sheep in Isfahan province. Many factors including climatic condition and presence of vector tick may be associated with the frequency of *A. ovis *infection in different areas.^25^

In contrast to this study, a high frequency of *A. ovis* was detected in the small ruminants in the east of Turkey where *Hyalomma *and *Rhipicephalus *species are dominant.^25^

In conclusion, our study identified a low prevalence of *A. ovis* in the blood of healthy sheep in West-Azerbaijan province. Although we did not find *A. marginale* in blood cattle of this area, additional studies with a larger sample size are required to determine the prevalence of infection in the cattle of West-Azerbaijan province.
